# On the brain-imaging markers of neural correlates of consciousness

**DOI:** 10.3389/fpsyg.2015.00868

**Published:** 2015-06-25

**Authors:** Talis Bachmann

**Affiliations:** Laboratory of Cognitive Neuroscience, Institute of Public Law, University of TartuTallinn, Estonia

**Keywords:** consciousness, neural correlates, contrastive analysis, contents of consciousness, criterion contents of report

For many years, since Baars (1988) explicitly formulated it, *contrastive analysis* has been the key methodological approach in experimental studies of consciousness. When certain properly chosen psychological experimental setups (allowing an invariant target stimulus either to be consciusly experienced or not) were combined with brain-imaging methods, contrastive analysis became a quite powerful tool of research (Crick, 1994; Koch, 2004). By subtracting markers of brain processes recorded in the conditions without conscious experience of the target from the markers recorded in the conditions where the same target is consciously experienced it was believed that the markers of neural correlates of consciousness (NCC) can be obtained. However, as it turned out in the subsequent theoretical and experimental analysis, the picture is not so clear and simple (Bachmann, 2000, 2009; Miller, 2007; Aru et al., 2012; de Graaf et al., 2012). For example, when in the invariant conditions of independent variables a masked visual stimulus was consciously perceived or not (consciousness of the target standed as a dependent variable), NCC which were measured as a spectral perturbation of EEG was present already *before* stimulus presentation (Aru and Bachmann, 2009). Thus, the neural correlate of consciousness of a stimulus was present earlier than the stimulus itself was presented. Now, a reader must not get excited here because instead of some paranormal explanations brain-science based explanations can be comfortably used.

In order to overcome the conceptual crisis hitting the traditional contrastive analysis based NCC research it was suggested that unconscious prerequisite processes (NCCpr) emerging as a result of contrastive analysis of brain-process markers of consciousness and similarly unconscious consequent processes (NCCco) must be differentiated from the constitutive processes directly associated with conscious experience (Aru et al., 2012; de Graaf et al., 2012). Thus, new experimental approaches were in need to avoid the trap of distilling prerequisite, direct, and consequent processes as mutually confounded and empirically inseparable. Despite some first attempts in this direction (Aru and Bachmann, 2015), the specialist landscape in this domain has remained obscure and no breakthrough solutions have been in sight. Moreover, there seems to be a number of additional uncertainties when we try to disentangle the various sub-types of NCC. Even NCCpr and NCCco are not unitary in terms of their theoretical meaning and associated neural processes. First, as the contents on which the perceptual report is founded can be selective, the markers of unused conscious contents may be erroneously neglected as markers of unconscious processes. They actually belong to consciousness level processes, but related to contents of consciousness qualitatively different from the ones specified by NCC. Second, in measuring NCC we must be able to disentangle contributions of the general consciousness enabling mechanisms and the selective contents representing mechanisms because their markers can be different and thus confused. In what follows I will substantiate these two issues.

## Variable criterion contents in reporting conscious experience

As stated above, NCCpr and NCCco may be incorrectly interpreted as markers of no-consciousness. From the perception research it is well known that one and the same stimulus (e.g., as a target in contrastive consciousness research) is experienced as a more or less complex combination of features and subjective image characteristics or attributes. Thus, reports about a stimulus by a subject are based on certain criteria of what to focus on for reporting the stimulus. In specialist literature the concept of *criterion contents* has been used for this (Kahneman, 1968; Jannati and Di Lollo, 2011; Sackur, 2013; Bachmann and Francis, 2014). This creates a situation where the contents defined by experimental instruction or intuitively applied by the subject may be different from the contents experienced before the stimulus as (a vague) expectation or after the stimulus as a (vague) immediate memory afterimage. This leads to the possibility that, while contents of NCCpr and/or contents of NCCco are different from direct NCC, the results of contrastive analysis will be incorrectly interpreted. Therefore, although actually there is some subjective content in experience before the stimulus or after the stimulus, due to the fact that according to the content this experience is different from the experience that characterizes direct NCC, NCCpr, or NCCco will be erroneously interpreted as markers of no-consciousness. Typically, a subject's experience in our type of experiments is this: before the target presentation he/she may have certain vague expectation of any stimulus, or expectation (spontaneous or pre-set) of some specific stimulus, which means there is certain epoch of stimulus-related conscious experience, albeit not as a response to an actual stimulus. Similarly, when the target has been briefly flashed (e.g., for 10 or 80 ms), a fleeting experience of its trace can linger for a second or more. Furthermore, even when after a moment the stimulus-related experience can fade to zero, subsequently when trying to come to the response the subject experiences a re-emerging (albeit vague) representation of the supposed stimulus in working memory. Thus, NCCco can be also indicative of consciousness as related to the target, but the contents of this conscious representation differ to some extent.

## General vs. specific markers

It is well known that brain systems necessarily involved in producing conscious experience can be classified as specific systems (SP) responsible for communicating and representing the *contents* of consciousness and non-specific (NSP) systems not carrying specific contents, but modulating the *level* of activity of the specific systems (reviews: Bachmann, 1994; Bachmann and Hudetz, 2014). As there is no consciousness without its contents (however indistinct or vague vs. clear or distinct it is) and because any content cannot be conscious without a sufficient level of activity of the NSP (interacting with SP), activity of both SP and NSP should in principle be able to contribute to the neural markers of consciousness in NCC. This by default creates an uncertainty with regard to what and to what extent contributes to the recorded markers of NCC. There is no known or definitely inevitableprecondition for objective markers of brain-imaging based NCC unambiguously ascribed to SP or NSP brain processes unless one can exhaustively measure the action of SP and NSP systems by appropriate neuroimaging methods. In most cases it is likely that brain-imaging results display contributions of NSP/SP interaction, but sometimes it can be exclusively SP (e.g., in its amplified mode) or NSP (e.g., as more ignited). Therefore, in order to advance our theoretical thinking for constraining experimental design or at least for sensible interpretation of empirical results we should first try at least conceptually to distinguish NCC/SP, NCC/NSP, and NCC/SP**×**NSP. The contribution of NSP is considered as a general marker invariant to specific contents. The need to use a tripartite NCC with regard to SP/NSP involvement comes from the respective neurobiological reality (Koch, 2004; Bachmann and Hudetz, 2014). If we really want to understand NCC, action of these subsystems can be and should be measured experimentally.

## The premises for a taxonomy

Based on the above considerations, we must think of NCC in terms of several possibilities: (i) NCCpr/general as related to the contribution of NSP which is reflected in brain-imaging markers and which appears as a result of contrastive analysis (NCCprG); (ii) NCC/general related to the contribution of NSP analogously to (i) (NCCG); (iii) NCCco/general related to the contribution of NSP analogously to (i) (NCCcoG). Similarly, with regard to SP we have: (iv) NCCprSP; (v) NCCSP; (vi) NCCcoSP. The further variants (vii), (viii), (ix) refer to NCCprG**×**SP, NCCG**×**SP, NCCcoG**×**SP, respectively. In what follows I will give some examples of the mental processes exemplifying the different items of this NCC taxonomy.

For NCCprG a typical situation involves attentional alerting necessarily preceding a target if a peri-threshold target is to become consciously perceived. It is possible that pre-target EEG phase contributes to this (Busch et al., 2009; Mathewson et al., 2009). NCCG might mean that neural markers of actual non-specific reticulo-thalamo-cortical modulation have been effectively recorded. The NCCG**×**SP, in turn, might mean that the markers of the processes recorded in the contrastive experiment reflect neural processes of interaction between active specific content-representing modules and non-specific reticulo-thalamo-cortical modulation. In the case of these taxons it would be both interesting and important to distinguish within-modal feature-specific modulation and pan-modal modulation universal for different sensory modalities. Thus, experiments are needed directly and selectively manipulating each of these effects (e.g., Pillay et al., 2014; Manita et al., 2015). In NCCcoG we might see a signature of a kind of “afterglow” of the NSP activity following a successful facilitative modulation of the specific contents-carrying neural units.

For NCCprSP the following are typical variants: (a) conscious expectancy of certain stimulus alternative before actual stimulus presentation, (b) pre-conscious priming of target-related neural nodes necessary for the subsequent successful target perception at the conscious level. (An interesting theoretical puzzle appears when we accept the possibility that the NCCpr process going on at the pre-conscious level can (and in many cases does) continue during the direct NCCSP when the target is directly perceived in consciousness. Should we term the same neural process now a constituent of consciousness (because it is present during conscious perception) or still a pre-requisite? By virtue of interaction with additional processes involved in NCC the formerly pre-requisite process may transform to a direct NCCSP process). Obviously, NCCSP signifies stimulus content-specific processes accompanying explicit conscious experience of the target stimulus. However, by itself, for NCCSP it is not clear whether it is brought about by exhaustive representation of target stimulus features and attributes, or only by a certain sub-sample or part of the whole. Especially here the notion of criterion contents becomes relevant. Future experiments should explicitly pay attention to the criterion contents as a purposely used variable in NCC research based on contrastive analysis. Moreover, according to the microgenetic stance of conscious perception every percept unfolds over successive stages where the subjective contents change in real time (Bachmann, 2000). Typically, coarse and less distinct stages precede more detailed and stable stages. When measurement of NCC is based on target responses indicative of the full-blown, stabilized target perception, the NCC indicative of an underdeveloped form and immature content of the target stimulus—and therefore still NCC—are erroneusly attributed to NCCpr. Understanding of this could help solve the controversies related to estimation of the time it takes to build up a conscious percept. It is highly likely that certain earlier signatures (e.g., ERP components N100, N200) as markers of the putative NCCpr are actually an early marker of NCC, but related to conscious contents of an immature stage of the percept under microgenetic development.

With NCCcoSP we have a similar problem like with other categories of NCC. Due to different qualitative contents of the immediate memory of the target stimulus compared to NCCSP, the NCCcoSP need not be a marker of unconscious consequences of the preceding conscious processing, but a marker of the temporally lingering and less distinct experience of the target when it is represented in the active working memory. On the other hand, NCCcoSP can be also a marker of some subliminal trace of the specific contents of the preceding target's conscious experience.

The above considerations stressing the relatively long duration of an experimental episode involving target awareness bring in a problem for interpreting, for example, the ERP or MEG markers of consciousness. Because the full microgenetic process of target conscious perception unfolds from proto-object, immature stage to a clear and detailed stage and thereafter the *seconds long* stage of immediate memory experience (which alltogether take several seconds as a minimum), markers such as visual awareness negativity (VAN) (Railo et al., 2011) whose wave spans barely a 100 ms and P300 whose wave takes a few 100 ms, are definitely inadequate to echo the real-time duration of the conscious experience related to the target stimulus. Instead, we may have cases of markers of *access* to certain level or stage of conscious processing, but not the one-to-one reflection of target-related conscious experience in real time.

We have to bear in mind that there are two types of the experimental criteria of subjective evaluation of contents depending on whether they are universal and possible to use panmodally (i.e., for evaluation of visual, auditory, tactile etc contents) or modality-specific. Attributes like “clarity,” “unity vs. fragmentedness,” “detailedness,” “duration,” “salience,” etc. can be used panmodally. The second type is specific to modality, such as brightness, color, slant, shape, edges/lines, loudness, timbre, phonemic composition, roughness, painfulness, temperature, etc. (of course, cross-modal and synaesthetic qualities can be also considered). Universal panmodal attributes of the consciousness contents are regulated by the brain systems responsible for the levels of consciousness (Bachmann, 2012). A recent fine example of how recording contrastive NCC by MEG can indicate differences in universal subjective evaluations is published by Andersen et al. (2015). Future experiments must directly disentangle contributions of the modality-specific content-systems and panmodal level-systems.

## Conclusions

The main messages of this opinion paper can be summarized as follows. Although, at first it may seem that brain-imaging results of contrastive anaysis indicate three types of NCC among which two (NCCpr and NCCco) refer to unconscious processes, a more careful analysis shows that in many cases these seemingly unconscious-process markers actually belong to consciousness level processes, albeit related to contents of consciousness qualitatively different from the ones specified by NCC. In trying to disentangle different subtypes of NCC, contributions of general, and content specific brain systems must also be differentiated.

### Conflict of interest statement

The author declares that the research was conducted in the absence of any commercial or financial relationships that could be construed as a potential conflict of interest.
